# Digital Follow‐Up After Total Knee Arthroplasty: An Interview Study Among Orthopaedic Surgeons

**DOI:** 10.1002/msc.70301

**Published:** 2026-11-02

**Authors:** Casper Craamer, Thomas Timmers, Walter van der Weegen, Rudolf Bertijn Kool

**Affiliations:** ^1^ IQ Health Science Department Radboud University Medical Center Nijmegen the Netherlands; ^2^ Research and Development Department Interactive Studios ‘s‐Hertogenbosch the Netherlands; ^3^ Department of Orthopedic Surgery Sports & Orthopedics Research Centre Anna Hospital Geldrop the Netherlands

**Keywords:** digital health, digital triage, orthopaedics, routine follow‐up, total knee arthroplasty

## Abstract

**Background:**

Traditionally, patients who undergo total knee arthroplasty (TKA) are scheduled for an in‐person routine follow‐up (RFU) regimen. Increasing pressure on orthopaedic services and growing acceptance of digital health technologies urge clinics to adopt more efficient RFU strategies. However, limited insight exists into how RFU after TKA is organised in clinical practice and under which conditions digital follow‐up is acceptable to orthopaedic surgeons. This study explored current RFU practices after TKA and examined surgeons’ perspectives on digital follow‐up.

**Methods:**

A qualitative study using semi‐structured interviews was conducted with 21 orthopaedic surgeons from different healthcare institutions. Interviews were conducted digitally, transcribed verbatim, and analysed using qualitative analysis software. Two researchers independently coded the transcripts, after which the codes were compared, refined, and grouped into overarching themes.

**Results:**

Four main themes emerged. (1) RFU after TKA was highly fragmented across institutions, with variation in timing, contact modality, responsible healthcare provider, and use of radiographic assessment. (2) Follow‐up visits were primarily perceived as a means to confirm uncomplicated recovery and detect complications early. (3) Digital follow‐up was viewed not as a replacement for in‐person visits but as an additional triage strategy to support decisions on physical consultation. (4) Acceptance of digital follow‐up depended on clear patient selection, predefined clinical recovery indicators, and preservation of professional and patient autonomy.

**Conclusion:**

RFU after TKA is poorly standardized and institution dependent. Digital follow‐up is considered acceptable mainly as a triage mechanism if patient safety, professional autonomy, and the surgeon–patient relationship are maintained.

## 1 Introduction

Traditionally, patients who undergo elective total knee arthroplasty (TKA) are scheduled for a routine follow‐up (RFU) regimen with the orthopaedic surgeon or a physician assistant. These RFU regimens typically consist of multiple scheduled outpatient visits in the weeks to years following surgery (e.g., at 2 weeks, 2 months, and annually thereafter). They are generally embedded in clinical guidelines (American Academy of Orthopaedic Surgeons 2022; Broughton et al. 2019; Dutch Orthopaedic Association 2021; National Institute for Health and Care Excellence 2020). This practice has been partly based on the rationale of identifying early postoperative complications—such as dislocation or infection—that may require further treatment and follow clinical recovery. However, such early complications are relatively rare and are hardly first identified during RFU visits, as symptomatic patients may request contact with their healthcare professional (HCP) regardless of scheduled follow‐up (Hart et al. 2021; Kingsbury et al. 2022). At the same time, the population is ageing with an associated increase in the prevalence of osteoarthritis (Inacio et al. 2017; Long et al. 2022; Matharu et al. 2022). The number of TKA procedures performed has substantially increased (Dutch Arthroplasy Register 2025; National Joint Registry 2025; Sloan et al. 2018), which urges outpatient orthopaedic clinics to look for opportunities to organise RFU more efficiently.

For more efficient RFU, an increasing number of digital opportunities are available. Patients are nowadays able to share their health status remotely through online questionnaires, mobile applications, or wearable devices allowing for continuous or episodic monitoring or structured self‐reporting outside the hospital setting (Ouendi et al. 2024; Plavoukou et al. 2025; Raje et al. 2024; Sierra et al. 2025). These tools could be used to support data‐driven clinical decision‐making in RFU, offering insight into recovery trajectories.

This raises the question whether these digital tools can provide sufficient information and reassurance for orthopaedic surgeons to reorganise the current RFU regimen. Little is known about the specific conditions under which orthopaedic surgeons would be willing to consider a shift from in‐person to digital follow‐up of TKA patients. It is important to know which data are deemed necessary to assess recovery, what adjustments in clinical workflows would be required, and how surgeons can feel sufficiently informed and confident to consider digital alternatives. Insight into these perspectives is crucial for a safe and acceptable transition to digital follow‐up regimens.

This qualitative study aims to explore the thoughts, expectations and views of orthopaedic surgeons on a transition from in‐person outpatient visits to digital follow‐up. This includes which types of digitally collected data are considered essential by surgeons and what data could support postoperative decision‐making in RFU. By identifying these perspectives, we aim to support the digital transformation towards data‐driven and need based models for RFU after TKA.

## 2 Method

### 2.1 Design and Participants

We conducted a qualitative study using semi‐structured interviews between June and November 2025. We invited orthopaedic surgeons who are involved in RFU of TKA patients within the outpatient clinic of academic centres, non‐academic teaching centres, general hospitals and private clinics. A purposive sampling strategy was used to capture variations in clinical experience and the institutional context. In addition, we used snowball sampling by inviting each surgeon at the end of the interview to nominate a colleague for study participation, thereby capturing even more diverse contexts and attitudes towards digital follow‐up.

### 2.2 Data Collection

Interviews were conducted through videoconferencing and were recorded and transcribed ad verbatim using Microsoft Teams software (Microsoft Corporation). Interviews were conducted by a trained qualitative interviewer (corresponding author [CC]) and supervised by a senior researcher (second [TT] and third [WvdW] author). Details of the research team are reported in Supporting Information S1: Multimedia Appendix 1. Participant characteristics and informed consent were collected at the beginning of the interview. Subsequently, in‐depth interviews were conducted, using a pre‐defined topic list (Supporting Information S1: Multimedia Appendix 2). Topics were based on existing literature and consequent discussions between the research team and included the following : (1) current RFU regimen, (2) perspectives on digital follow‐up, (3) needs for clinical decision making, and (4) conditions for implementation.

Data collection continued until thematic saturation, defined as no new themes or mechanisms emerging in the two subsequent interviews. Interview summaries and a summary of the findings were returned to participants for member checking to enhance credibility, correct errors, clarify interpretations, and allow for additional input (O'Brien et al. 2014). Finally, the interviews were anonymised.

### 2.3 Data Analysis

An initial open coding phase was conducted across the first three transcripts by two independent researchers (CC and TT). Codes were developed inductively from the interview transcripts and iteratively refined through comparison across transcripts until consensus was reached about a preliminary codebook. Each code was explicitly defined, with inclusion and exclusion criteria specified to ensure consistent application (Supporting Information S1: Multimedia Appendix 3). This codebook was then used by the primary researcher (CC) to code the transcripts. If new codes emerged, all interviews were reviewed according to the new code. ATLAS.ti (version 24.0.0.29576) was used to support the coding process. All quotations were retained verbatim to preserve analytic integrity and subsequently translated into English. Semantic accuracy of the translated quotations was ensured through review by the research team and back translation. Coding focused on identifying explicit statements. Codes were grouped into candidate themes through constant comparison across interviews. A cross‐case saturation matrix was constructed to assess the presence and depth of themes across participants (Supporting Information S1: Multimedia Appendix 4). The Consolidated criteria for reporting qualitative research (COREQ) was used for reporting (Tong et al. 2007).

## 3 Ethical Approval

The Medical Review Ethics Committee concluded that this study was not subject to the Medical Research Involving Human Subjects Act and approved the study (case number 2025‐18164). All participants gave digitally informed consent prior to the interviews.

## 4 Results

### 4.1 Participant Characteristics

Between June and November 2025, 21 orthopaedic surgeons were interviewed. Most of them were male (*n* = 20, 95.2%). Participants ranged in working experience from 2 to 25 years (mean 11.3 SD 6.2). Interviews ranged in duration from 21 to 48 minutes (mean 36 SD 6.4). Participant and interview characteristics are reported in Supporting Information S1: Multimedia Appendix 5.

### 4.2 Main Themes

From the interview analysis, four themes were identified in the early TKA period: (1) fragmented RFU regimens between care settings, (2) various values of follow‐up visits, (3) digital follow‐up conceptualised as a triage mechanism, and (4) acceptance of digital follow‐up is conditional. Subthemes for each theme were identified (Table 1).

**TABLE 1:** Identified themes and subthemes.

Theme	Subtheme	Explanation
Fragmented routine follow‐up regimens between care settings	Timing	The timing and frequency of postoperative follow‐up, including early, intermediate, and long‐term contacts.
	Modality	The way in which follow‐up is delivered, including in‐person, telephone, and digital approaches.
	Involved clinician	Which healthcare professionals are involved in follow‐up across settings and moments.
	Use of radiographic imaging	The use and perceived necessity of radiographic imaging during follow‐up.
Various values of follow‐up visits	Clinical value	Perceived usefulness of follow‐up visits and contacts
	Outcome assessment	Assessment of recovery during follow‐up contacts
	Reassurance and relational value	Role of routine follow‐up in reassurance, expectation management, and maintaining the care relationship.
Digital follow‐up conceptualised as triage mechanism	Potential role and data sources	Use of digital follow‐up to monitor recovery and identify deviations using selected data sources.
	Perceived concerns and risks	Concerns about safety, data reliability, and missed clinical issues with digital follow‐up.
Acceptance of digital follow‐up is conditional	Patient selection	Criteria used to determine which patients are suitable for a potential digital follow‐up.
	Clinical decision rules	Use of structured criteria to guide triage and escalation decisions.
	Clinician and patient autonomy	Importance of preserving clinician judgement and patient choice in follow‐up decisions.

### 4.3 Theme 1: Fragmented Routine Follow‐Up Regimens Between Care Settings

Across interviews, participants described substantial variation in postoperative RFU. RFU differed markedly between institutions with respect to timing, modality, involved HCP, and use of radiographic imaging.

#### 4.3.1 Timing

The timing of a first TKA follow‐up visit varied with participants reporting an early wound check at approximately 2 weeks to a first contact around 6–8 weeks that included physical examination and radiographic evaluation. Long‐term follow‐up practices differed even more. Some participants reported routine 1 and 5‐year follow‐up visits, while others described a shift away from traditional long‐term controls with a reduced frequency or even absence of these in‐person follow‐up visits, if recovery proceeded without complications.

In our practice, we do a follow‐up after eight weeks. And then at 12 months and five years. Unless the patient is older than 80 by then. In that case, we discuss it and say: only come back if you have complaints.

(ID3)

#### 4.3.2 Modality

Modality of RFU was equally heterogeneous. RFU is conducted in‐person at the outpatient clinic, by telephone, or—less frequently—through digital monitoring.

After a few months, an X‐ray is taken at one of our locations. After that, I have telephone contact with the patient. Previously, there was also a follow‐up after one year and after ten years, both by telephone, but we are phasing those out.

(ID1)

#### 4.3.3 Involved Clinician

There was a wide range of participants who were involved in RFU. RFUs were variously nurse‐led, conducted by specialised nurses, physician assistants, sometimes by residents, or the operating surgeon. Participants emphasised that direct involvement of the operating surgeon was primarily required when recovery deviated from expectations or when clinical decision‐making was necessary.

That could certainly also be done by a nurse specialist. That follow‐up does not necessarily have to be carried out by me as a surgeon.

(ID2)

#### 4.3.4 Use of Radiographic Imaging

The use and timing of radiographic imaging further illustrated this fragmentation. Some participants described routine postoperative imaging followed by additional radiographs at 1 year, while others considered routine radiographs as low added value and rarely performed these unless clinically indicated.

It is very rare that I see anything different on the six‐week X‐ray compared to the X‐ray taken during admission. I do not practice ‘radiographic medicine’. For that reason, I feel confident about letting go of the X‐ray.

(ID20)

### 4.4 Theme 2: Various Values of RFU

#### 4.4.1 Clinical Value

Participants described that from a surgeon perspective, outpatient RFU often has limited interventional value for patients with an uncomplicated recovery. Instead, its value lay in confirming that recovery was progressing as expected. In contrast, for patients with a deviated recovery, follow‐up visits may identify early complications.

About ten years ago, I even received a complaint from a young female patient who felt she had to come for a six‐week follow‐up unnecessarily, even though everything was going well. At the time, we thought that was ridiculous, but that woman indicated that she had a busy job and life and therefore questioned why she had to come to the hospital, given that the recovery was going well.

(ID12)

#### 4.4.2 Outcome Assessment

From an outcome perspective, surgeons consistently reported a prioritised combination of subjective and objective domains. Subjectively, they focused on pain management and patient satisfaction. Objectively, assessment cantered on wound healing and knee function, particularly range of motion (ROM). A few participants noted that simply observing how a patient walked into the consultation room provided valuable information that could not easily be captured differently.

I always ask how the wound has healed. I also want to know whether they have stopped using their assistive devices and whether they have had physiotherapy. What I also ask is whether they are still using pain medication; I consider that important. Then I perform a physical examination. I also want to know how far the patient can bend and extend.

(ID17)

#### 4.4.3 Reassurance and Relational Value

Beyond clinical outcomes, participants emphasised the importance of reassurance, expectation management, and relational continuity. Even when no changes in care management were required, the visit served to confirm for both patient and clinician that recovery was on track.

I do think it is pleasant for the patient to have contact with the healthcare provider after the operation, so that they feel they are being properly supported.

(ID15)

### 4.5 Theme 3: Digital Follow‐Up Conceptualised as Triage Mechanism

Across interviews, digital follow‐up was rarely framed as a full substitute for in‐person care. Instead, it was conceptualised as a means to monitor recovery trajectories, detect deviations from expected progressand, subsequently, triage who needed in‐person care.

#### 4.5.1 Potential Role and Data Sources

Overall, participants expressed cautious optimism about relying solely on patient‐reported data to monitor recovery trajectories. Most respondents described that patients could provide accurate answers at the comfort of their homes when prompted to answer questions regarding their recovery. Other types of data were mentioned as added value for decision‐making, such as observations reported from allied physiotherapist, tracking data or photo and videographic data. Nevertheless, many participants acknowledged that digital tools could play a valuable role in identifying patients who were not recovering as expected with its potential to triage who needed a follow‐up visit.

Digital tools should be used to detect deviations, not to keep everyone away.

(ID19)

#### 4.5.2 Perceived Concerns and Risks

In rare cases, some concerns were raised about the risk of missing complications, particularly subtle wound problems or delayed functional recovery, if digital follow‐up was implemented without adequate safeguards. Some of these participants nuanced their assumptions themselves by noting that such complications are relatively rare and symptomatic patients may request contact with their HCP regardless.

Additionally, a few participants noted that reduced RFU could limit learning opportunities for early‐career surgeons. In addition, outpatient visits could become more complex, as only those with complications or poor recovery present to the clinic.

Young doctors miss out on a learning curve. It is important to know what the impact of the operation is during the first two months for a patient.

(ID18)

### 4.6 Theme 4: Acceptance of Digital Follow‐Up is Conditional

Acceptance of digital follow‐up was explicitly conditional. Participants emphasised the need for clear patient selection criteria, reliable escalation mechanisms, and the ability for clinicians and patients to override digital protocols at any time.

#### 4.6.1 Patient Selection

Several clinicians described a ‘digitally first, unless’ approach, in which digital follow‐up could be the default for digitally skilled patients with an uncomplicated recovery, but in‐person review remained readily accessible based on data‐driven alerts, clinical judgement, or patient preference.

First of all, patients need to be digitally skilled. They need to be able to take a photo independently and send it.

(ID9)

#### 4.6.2 Clinical Decision Rules

Many participants favoured a simple threshold‐based triage approach: if patient‐reported data meet predefined criteria, the outpatient visit is medically considered unnecessary. Participants agreed that clinically meaningful decision‐making is supported by capturing structured data. The key recovery indicators identified were: (1) knee function (2) wound condition, and (3) pain level. Knee function is considered normal at flexion > 90° and extension ≤ 5°, the wound should be closed and dry, and adequate analgesia is applied with discontinued opioid use to keep pain level under control.

If one of the three elements is not considered ‘green’—functional recovery, clinical relevance, and patient preference—the patient is more than welcome.

(ID4)

Several participants suggested that these data could be captured through structured patient self‐reports on progress. Others mentioned that a physiotherapist could be involved in reporting ROM due to concerns for inaccurate patient reporting, or patients could be asked whether they can cycle on a home trainer as a proxy for sufficient flexion.

A few participants advocated for serial measurements beginning in the first postoperative week to capture recovery trajectories, though others warned that this might yield false positives due to normal variations in early pain and function. Consensus existed that triage should not occur in the immediate postoperative phase, when complications are more common and recovery is highly variable.

#### 4.6.3 Patient and Clinician Autonomy

Patient access to care was considered essential. Participants stressed that patients must be explicitly informed that they could always return for evaluation, even long after surgery, if concerns arise.

Loss of professional autonomy or relational continuity was perceived as a barrier to adoption. Digital follow‐up was rejected when it was seen as constraining clinical judgement or diminishing the doctor–patient relationship. Similarly, preserving patient autonomy emerged as a key principle. Even if algorithmic assessment suggests no need for review, patients should retain the option to request in‐person visitation.

For example: ‘Based on your answers, everything appears to be going well. Would you like to cancel the appointment?’ Then you click yes or no.

(ID5)

## 5 Discussion

### 5.1 Principal Findings

This qualitative study explored how RFU after TKA is currently organised and under which conditions digital follow‐up would be considered acceptable in optimising or replacing RFU. In total, 21 orthopaedic surgeons were interviewed from which four main themes emerged. First, participants demonstrated that, despite clinical guidelines, RFU regimens varied markedly across healthcare institutions with respect to timing, modality, involved HCP, outcome assessment and use of radiographic imaging. Second, participants addressed several values for follow‐up visits, but primarily served to distinguish uncomplicated from deviating recovery. Third, participants did not consider digital follow‐up as a replacement for existing RFU regimens. Instead, it was regarded as a context‐sensitive triage mechanism, aimed at identifying deviations from the expected recovery trajectories and determining which patients required in‐person care. Fourth, the acceptance of digital follow‐up was explicitly conditional.

Overall, the themes are related to one another. The fragmentation in Theme 1 indicates that RFU is based on local routine rather than scientific consensus, which may explain participants’ willingness to reconsider current practice. The relational value of in‐person follow‐up, addressed in Theme 2, may explain why digital follow‐up was regarded as a triage mechanism rather than a replacement (Theme 3), thereby reserving in‐person contact for those who need it. Moreover, participants agreed on the key recovery indicators (Theme 4), but differed on timing and whether to rely solely on patient reported data.

Orthopaedic surgeons were open to digitally supported triage mechanisms under the condition that tools should collect meaningful information on various domains, including knee function, wound condition, and pain control. Thresholds should be defined and clear escalation mechanisms should be in place to distinguish uncomplicated from deviating recovery. In addition, irrespective of triage outcomes, both surgeons and patients were considered to retain the discretion to request an in‐person consultation when deemed necessary. Consensus existed that triage based on these indicators should not occur before 6 weeks after surgery, as early recovery was considered highly variable and complications were more frequent during this phase.

The findings of a heterogenous RFU landscape align with existing literature and are demonstrated in fracture care (Coeckelberghs et al. 2024) and chronic care (Stephenson et al. 2019) as well. In addition to this alignment, acceptance of digitalisation in orthopaedic care has also been demonstrated earlier (Chang et al. 2025; Craamer et al. 2024, 2025; Timmers et al. 2019). Nevertheless, resistance emerged when digital follow‐up was perceived to constrain clinical judgement or undermine the doctor–patient relationship. Similarly, maintaining patient choice was considered essential, reflecting broader evidence that a substantial proportion of patients continue to value in‐person consultation even when digital follow‐up options are available (Hafeez et al. 2022; Li et al. 2022).

This preference for in‐person contact also raises the question of how patients engage with digital alternatives once they are offered. A survey among patients using a smartphone‐based care management platform following robotic TKA reported overall appreciation of the application. It also identified limitations in its actual use and in patients’ perception of its value, emphasising the need for structured recruitment, adherence strategies, and integration of digital tools into a comprehensive patient journey (Rossi et al. 2024). This suggests that the conditions identified by orthopaedic surgeons, such as careful patient selection and preservation of patient autonomy, are necessary but not sufficient. Successful implementation of digital follow‐up will equally depend on sustained patient adherence and on embedding digital tools within the broader care pathway rather than offering them as stand‐alone solutions.

The consensus that triage should not occur before 6 weeks after surgery, seems reasonable due to considerable variations of recovery trajectories between individuals (Dumenci et al. 2019). Beyond timing, the acceptability of digital triage appeared to depend on the perceived reliability of the underlying data. Surgeons in the present study expressed greater confidence in digital follow‐up when patient‐reported data were complemented by physiotherapist observations or visual materials such as photographs or video. Studies in telemonitoring across other clinical domains similarly suggest that combining patient‐reported outcomes with professional assessment or objective inputs enhances monitoring reliability compared with patient self‐report alone (Gelbman and Reed 2022; Sierra et al. 2025). Notably, part of this need for objective verification may be addressed by existing technology. A review of remote monitoring after TKA reported that smartphone applications and wearable devices can capture accurate and reproducible data on knee function and activity, allowing HCPs to identify patients who deviate from the expected recovery trajectory (Sodhi et al. 2023). These technologies thus align with the triage concept articulated by participants in the present study, particularly for functional recovery indicators, although their integration into clinical workflows remains a prerequisite for adoption. Finally, while concerns were raised about the possibility of missing complications when relying on digital monitoring, these concerns were nuanced rather than absolute. Participants emphasised that many postoperative complications are symptomatic and would likely prompt patients to seek care regardless of formal RFU schedules, an observation that is consistent with existing literature (Hart et al. 2021; Kingsbury et al. 2022).

### 5.2 Strengths and Limitations

A key strength of this study is the inclusion of orthopaedic surgeons from a wide range of institutional contexts, including academic centres, non‐academic teaching hospitals, general hospitals, and private clinics. This diversity enabled the identification of themes that cut across heterogeneous local practices, strengthening the transferability of findings. In addition, methodological rigour was supported through independent dual coding, a structured codebook with explicit inclusion and exclusion criteria, and systematic saturation assessment using a cross‐case matrix.

Several limitations should be addressed. First, the sample was composed of orthopaedic surgeons, with no representation of physician assistants, specialised nurses, or residents. As these professionals are often involved in the RFU in daily practice as well, their perspectives on feasibility and workflow integration may differ from those of surgeons. This may have influenced the findings towards a physician‐centred view on digital follow‐up and its implementation within existing hospital workflows. Second, while this study provides insight into perceived implications of digital follow‐up, it does not empirically assess its impact on hospital workflow, such as changes in workload, coordination, or resource allocation. Third, this study focused exclusively on HCP perspectives. Patient views on the acceptability and perceived safety of digital follow‐up were not explored and may differ substantially from HCP assumptions. Finally, the study focused specifically on TKA follow‐up. Therefore, findings may not be directly transferable to other orthopaedic procedures with different population‐ and complication profiles or recovery trajectories.

### 5.3 Future Research

Future studies should focus on exploring patient perspectives on digital follow‐up in TKA. While orthopaedic surgeons expressed assumptions about patient preferences regarding digital follow‐up, these assumptions have not been validated. Qualitative and quantitative studies among TKA patients are needed to assess their perspectives to engage with digital triage mechanisms, their informational needs during recovery, and the conditions under which they would find digital follow‐up acceptable and reassuring. Furthermore, it is important for future studies to evaluate if implementing digital follow‐up tools reduces the burden on health care, and to what financial costs.

In addition, the clinical decision rules that emerged from this study—specifically the combination of adequate knee function, a closed and dry wound, and controlled pain without opioid use—provide a concrete foundation for further operationalisation. Future studies should develop and validate a structured digital triage mechanism based on these criteria and evaluate its clinical utility. Finally, the optimal timing for digital triage requires further investigation. Although participants reached a consensus that triage should not occur before 6 weeks postoperatively, this threshold was based on early recovery variability. Analysis of recovery trajectories using longitudinal patient‐reported outcome data could help identify the earliest safe and informative moment for digital assessment.

### 5.4 Practical Implications

The findings of this study carry several implications for the design and implementation of digital follow‐up after TKA. First, and most fundamentally, this study documented substantial variation in RFU regimens across institutions—in timing, modality, involved clinician, outcome assessment and use of radiographic imaging. This heterogeneity implies that digital follow‐up solutions cannot be designed as rigid, one‐size‐fits‐all protocols. Rather, any digital triage tool must offer sufficient flexibility to align with local workflows, institutional preferences, and existing care pathways. Second, the finding that several surgeons valued physiotherapist input as complementary to patient self‐reporting highlights the potential for cross‐professional digital collaboration. Implementing structured reporting mechanisms between physiotherapists and orthopaedic teams could strengthen the reliability of digital triage and create a more comprehensive picture of patient recovery without requiring an in‐person orthopaedic visit. Finally, the ‘digitally first, unless’ approach that is identified, relies on patients' digital literacy. Since the TKA population has predominantly a higher age, patients may have limited access to suitable devices and internet access, language support, or little digital confidence (Yao et al. 2022). Therefore, digital literacy should not be a neutral selection criterion but may exclude a significant proportion of patients. It might be better to provide a digital follow‐up optionally, with telephone or in‐person follow‐up remaining available, and HCPs or relatives could support patients with digital reporting.

## 6 Conclusion

In conclusion, RFU after TKA is shaped by institution‐dependent practices. Orthopaedic surgeons accept digital postoperative follow‐up not as a replacement for care, but as a conditional triage mechanism. Acceptance depends on whether digital follow‐up preserves patient safety, professional and patient autonomy, and the relational dimensions of care. This study provides insight into the specific conditions under which surgeons consider digital follow‐up acceptable, including the types of clinical data required and the importance of alignment with existing workflows. These findings highlight the need for flexible, context‐sensitive implementation strategies rather than standardised solutions. Future research should focus on incorporating patient perspectives, validating digital triage criteria, and evaluating the impact of digital follow‐up on healthcare utilization, costs, and clinical outcomes. Implementation efforts that fail to address these conditions are likely to encounter resistance, regardless of technological sophistication.

## Author Contributions

**Casper Craamer:** conceptualization, formal analysis, investigation, methodology, project administration, writing – original draft preparation, writing – review and editing. **Thomas Timmers:** conceptualization, formal analysis, methodology, supervision, validation, writing – review and editing. **Walter van der Weegen:** conceptualization, methodology, supervision, validation, writing – review and editing. **Rudolf Bertijn Kool:** conceptualization, methodology, supervision, validation, writing – review and editing.

## Funding

The authors have nothing to report.

## Conflicts of Interest

Casper Craamer and Thomas Timmers are employed in the research and development department of Interactive Studios. Interactive Studios is a company that develops a smartphone app that can be used for telemonitoring. The other coauthors declare that the research was conducted in the absence of any other commercial or financial relationships that could be construed as a potential conflict of interest. Moreover, all authors have completed the ICMJE uniform disclosure form and declare the following: no support from any organization for the submitted work, no financial relationships with any organizations that might have an interest in the submitted work in the previous 3 years, and no other relationships or activities that could appear to have influenced the submitted work.

## Supporting information


Supporting Information S1

## Data Availability

The data that support the findings of this study are available from the corresponding author upon reasonable request.
