# Supplementary material for: Digital Follow‐Up After Total Knee Arthroplasty: An Interview Study Among Orthopaedic Surgeons
Source: Musculoskeletal Care. 2026 Nov 2;24(4):e70301. doi: 10.1002/msc.70301 (PMC13727082; doi:10.1002/msc.70301)
Supplement: Supplementary file 1 [file MSC-24-e70301-s001.docx]

## Multimedia Appendix

### 1. Details of the research team

Below are more details of the team who conducted the research.

| Personal Characteristics | Primary researcher | Secondary researcher 1 | Secondary researcher 2 |
| --- | --- | --- | --- |
| Initials | CC | TT | WvdW |
| Interviewer/facilitator | Conducted interviews | Supervised interviews | Supervised interviews |
| Credentials | Msc | PhD | PhD |
| Occupation | Phd-Candidate | Post-doc | Post-doc |
| Gender | Male | Male | Male |
| Experience and training | Trained with basic experience | Advanced | Advanced |
| Participant knowledge of the interviewer | Conscious of personal goal to pursue a PhD | Conscious of co-supervising CC | Conscious of co-supervising CC |
| Interviewer characteristics | PhD-Candidate | Co-supervisor of CC | Co-supervisor of CC |

### 2. Topic list with optional questions to guide the interview

#### Start

- Thank you for your willingness to participate
- Introduction of the interviewer
- Introduction of the project
- Audio recording and consent forms

#### Topic 1: Current RFU regmine

1. What does the post-operative follow-up protocol look like in your clinic?
   - Modality: telephone, digital, in-person
   - X-ray?
   - Wound assessment
2. What data/information do you collect to assess the patient’s recovery?
   - Objective and/or subjective data?
3. How important is it for you to see the patient in person during this process?
   - Why is that?

#### Topic 2: Perspectives on digital follow-up

1. How would you feel if the patient were not seen in person for this post-operative follow-up?
2. What are your views or experiences regarding digital follow-up?
   - What do you see as advantages for yourself as a healthcare provider? And for the patient?
   - What do you see as disadvantages for yourself as a healthcare provider? And for the patient?
   - What is your opinion regarding the potential of missing other types of complications? Could these be asymptomatic?

#### Topic 3: Needs for Clinical Decision-Making

1. What data/information would you need to be able to make clinical decisions remotely?
   - For example: pain score, mobility, activities of daily living (ADL) functioning, swelling, redness, temperature, patient satisfaction
2. How many weeks after surgery would you first want to conduct such a digital assessment?
   - How often would you want to conduct a digital assessment?
   - At what point would you reconsider and opt for an in-person visit?
3. What would be a concrete reason for you to decide that an in-person visit is not necessary?
4. What would be a concrete reason for you to decide that an in-person visit is necessary?

#### Topic 4: Conditions for implementation

1. What would be required to make this feasible in your practice?
2. Who do you believe should be responsible for evaluating the data?

#### Closing

1. Are there any additional considerations you would like to share with us?
2. Do you have suggestions for other colleagues we could speak with about this topic?

### 3. Codebook

#### Theme 1 – Fragmented routine follow-up regimens between care settings

##### Subtheme 1.1 – Timing

**Code:** Followup_schedule_description

**Description:** Descriptions of the timing, frequency, and sequence of routine postoperative follow-up moments after TKA.

**Include when:** Interviewee describes when patients are seen or contactedMentions specific time points (e.g. 2 weeks, 6–8 weeks, 1 year)

**Exclude when:** Focus is on what is assessed during the visit (→ Theme 2)

**Code:** Timing_two_week_control

**Description:** References to wound checks, suture removal, or early postoperative controls around two weeks.

**Include when:** Explicit mention of “two weeks” or early wound check

**Exclude when:** General early recovery talk without timing

**Code:** Timing_six_to_eight_week_control

**Description:** Mentions of routine six-to-eight week postoperative follow-up.

**Include when:** Discussed as standard practice, debated, or removed

**Exclude when:** 6-8 week outcomes discussed abstractly (→ Theme 3 or 4)

**Code:** Timing_three_month_control

**Description:** Mentions of routine three month postoperative follow-up.

**Include when:** Discussed as standard practice, debated, or removed

**Exclude when:** 3 month outcomes discussed abstractly (→ Theme 3 or 4)

**Code:** Timing_one_year_control

**Description:** Mentions of routine one-year postoperative follow-up.

**Include when:** Discussed as standard practice, debated, or removed

**Exclude when:** One-year outcomes discussed abstractly (→ Theme 3 or 4)

**Code:** Reduction_of_routine_controls

**Description:** Definition Explicit statements about reducing, abolishing, or questioning routine follow-up visits.

**Include when:** Follow-up is described as stopped, shortened, or minimized The reason given is low yield, tradition, or efficiency

**Exclude when:** Reduction is conditional on digital tools (Theme 3/4)

##### Subtheme 1.2 – Modality

**Code:** In_person_followup

**Description:** "References to follow-up care delivered in person at the outpatient clinic, including explicit mentions of face-to-face visits as the standard, preference, or benchmark against which other modalities (e.g., phone, app-based) are compared.

**Include when:** Interviewee mentions physical/outpatient/in-clinic contact as the default or preferred mode of follow-up; Statements contrast in-person visits with digital or telephone alternatives; In-person follow-up is cited as necessary for physical examination, reassurance, or interpersonal reasons

**Exclude when:** Statements focus on the digital alternatives themselves (→ Telephone_followup, App_based_followup, Virtual_care_platform); In-person visit is mentioned only as part of imaging needs (→ Use_of_xray, Complex_cases_need_physical_visit); Focus is on who provides the care, not how it is delivered (→ Followup_by_... codes)"

**Code:** Telephone_followup

**Description:** Follow-up conducted by phone rather than in person.

**Include when:** Telephone replaces or complements physical visits

**Exclude when:** Calls discussed only as administrative

**Code:** App_based_followup

**Description:** Follow-up conducted by smartphone application rather than in person.

**Include when:** smartphone application replaces or complements physical visits

**Exclude when:** apps discussed beyond follow-up posibilities

**Code:** PROMs_based_followup:

**Description:** Use of PROMs or questionnaires in follow-up.

**Include when:** PROMs are mentioned for monitoring or screening; Benefits or limits are discussed.

**Exclude when:** PROMs are used only for research or benchmarking

**Code:** Virtual_care_platform

**Description:** Mentions of apps, portals, digital platforms, or structured digital systems used for follow-up (e.g., patient journey app).

**Include when:** Any named or described digital tool/platform used to monitor recovery or collect data; Discussion includes logging in, interface, app workflow.

**Exclude when:** Paper PROMs or generic “questionnaires” without platform context (use PROMs_based_followup); Wearables/sensors discussion (use Skepticism_toward_sensors etc.)

**Code:** Followup_changed_over_time

**Description:** Statements describing how follow-up practice has changed (reduced, shifted to phone, removed x-rays, etc.) across time.

**Include when:** Interviewee contrasts past vs current regimen (“vroeger… nu…”); Mentions a transition such as dropping one-year controls or changing who conducts follow-up.

**Exclude when:** Change is explicitly attributed to COVID-19 (use Impact_of_COVID_on_followup); The focus is on future implementation conditions (Theme 4)

**Code:** Impact_of_COVID_on_followup

**Description:** Statements attributing follow-up changes to COVID-19 (e.g., shift to phone, reduced clinic visits).

**Include when:** COVID is named explicitly as cause/context for changing follow-up modality or frequency.

**Exclude when:** Generic efficiency or capacity pressures are given without COVID reference (Theme 4: Efficiency_pressure); Long-term trend is described without COVID as driver (use Followup_changed_over_time)

##### Subtheme 1.3 – Involved clinician

**Code:** Followup_by_PA_or_NP

**Description:** Follow-up conducted by physician assistants, nurse practitioners, or nursing staff.

**Include when:** Task substitution is described

**Exclude when:** Digital or automated follow-up without human intermediary

**Code:** Followup_by_orthopedic_surgeon

**Description:** Follow-up conducted by orthopedic surgeon.

**Include when:** Task substitution is described

**Exclude when:** Digital or automated follow-up without human intermediary

**Code:** Followup_by_nurse_or_assistant

**Description:** Follow-up conducted by nurse or assistant.

**Include when:** Task substitution is described

**Exclude when:** Digital or automated follow-up without human intermediary

**Code:** Followup_by_general_practitioner

**Description:** Follow-up conducted by general practitioner.

**Include when:** Task substitution is described

**Exclude when:** Digital or automated follow-up without human intermediary

##### Subtheme 1.4 – Use of radiographic imaging

**Code:** Xray_as_routine

**Description:** Statements that x-rays are routinely performed at a certain follow-up moment (e.g., 6–8 weeks or 1 year), regardless of symptoms.

**Include when:** “standard photo at…”; Imaging described as embedded in protocol.

**Exclude when**: X-ray is done only when symptoms/indications exist (use Limited_value_of_xray if framed as low yield or “only on indication” logic); Imaging is discussed as essential condition for digital follow-up (Theme 4: may also code Complex_cases_need_physical_visit if linked to inability to assess fixation remotely)

**Code:** Limited_value_of_xray

**Description:** Statements framing routine x-rays as low-yield, rarely informative, or unnecessary unless clinically indicated.

**Include when:** “rarely see something new”, “no added value”, “not do fotogeneeskunde”, “only on indication”.

**Exclude when:** General reductions in follow-up without imaging focus (Reduced_clinic_visits); Imaging discussed purely descriptively without evaluative claim (use Use_of_xray)

**Code:** Use_of_xray

**Description:** Use of imaging as part of follow-up.

**Include when:** X-rays are discussed as routine or on indicationTiming of imaging is specified

**Exclude when:** Imaging is discussed as a barrier to digital follow-up (Theme 4)

**Code:** Xray_as_reassurance

**Description:** Imaging is used communicatively/educationally during the visit.

**Include when:** Xray is used for reassurance understanding reasons.

##### Subtheme 1.5 – Transcending the theme

**Code:** Variation_between_hospitals

**Description:** Follow-up practices justified by “variations within hospital” rather than evidence.

**Include when:** Interviewee explicitly questions rationale; Refers to others within the hospital who does it differently

**Exclude when:** Evidence-based justification is provided

**Code:** Reduced_clinic_visits

**Description:** Explicit mention of decreasing the number of in-person clinic visits (regardless of whether replaced by phone/app/on-demand).

**Include when:** Statements like “we see them less”, “we reduced controls”, “we don’t do routine visits anymore”; Reduction is framed as an outcome of a policy/practice decision.

**Exclude when:** The focus is why reduction is acceptable (Theme 2: Low_clinical_yield_visit); The focus is how reduction is implemented with safeguards (Theme 4: Clinical_override_protocol, Patient_selection_criteria)

**Code:** Guideline_influenced_practice

**Description:** References to professional guidelines (e.g., NOV/orthopaedic guidance) shaping follow-up frequency, timing, or imaging.

**Include when:** Interviewee explicitly mentions a guideline, society recommendation, or mandated follow-up intervals; Guideline is framed as reason to keep/perform controls or imaging.

**Exclude when:** Practice is framed as “always done” without guideline reference (use Visit_as_tradition); Evidence/trials are discussed (Theme 4: Proof_before_implementation / Randomised_trials_needed)

#### Theme 2 – Various values of follow-up visits

##### Subtheme 2.1 – Clinical value

**Code:** Detection_of_complications

**Description:** Identification of postoperative complications.

**Include when:** Infection, thrombosis, stiffness, or fixation issues are mentioned; Follow-up is framed as complication detection.

**Exclude when:** Complication detection is automated/digital (Theme 3)

**Code:** Low_clinical_yield_visit

**Description:** Definition: Statements indicating that routine visits rarely lead to intervention or change in management.

**Include when:** Visit described as reassurance or confirmation only

**Exclude when:** Visit described as essential for complication detection

**Code:** Uncomplicated_recovery

**Description:** Statements describing a normal, expected recovery course without complications; often used to justify reduced follow-up.

**Include when:** “if everything goes well”, “the majority goes fine”, “uncomplicated”; Used as reference category against which deviations are identified.

**Exclude when:** Recovery is described as off-track or delayed (use Deviating_recovery); Statements about eligibility for digital follow-up without explicit recovery quality (Theme 4: Low_risk_patients)

**Code:** Deviating_recovery

**Description:** Statements about recovery not proceeding as expected (delayed ROM, persistent pain, wound issues) and prompting additional assessment or visits.

**Include when:** “not going well”, “behind schedule”, “outliers”, “red flags”, “stiffness”; Links deviation to triage/need for in-person review.

**Exclude when:** Specific complication detection without trajectory framing (use Detection_of_complications); Purely threshold-based selection criteria without narrative of deviation (Theme 4: Patient_selection_criteria)

**Code:** Gut_feeling_clinician

**Description:** Statements indicating a clinician’s intuitive judgment (“pluis/niet pluis”, gut feeling) guiding follow-up decisions.

**Include when:** Clinician describes relying on intuition or overall impression not reducible to metrics.

**Exclude when:** The clinician explicitly grounds decisions in years of experience or pattern recognition (use Experience_based_assessment); Decisions are based on data thresholds (Theme 4: Data_driven_decision_making)

**Code:** Eyeballing_patient

**Description:** Tacit visual or experiential assessment of patients.

**Include when:** Clinicians describe “seeing how someone walks”; Intuitive or observational assessment is mentioned

**Exclude when:** Assessment is formalized or protocolized

**Code:** Experience_based_assessment

**Description:** Statements showing decisions are based on accumulated clinical experience, pattern recognition, or “what I’ve seen over years”.

**Include when:** “With experience you see…”, “in my experience…”, “after years you learn…”; Assessment described as learned expertise rather than formal criteria.

**Exclude when:** Intuition without reference to experience (use Gut_feeling_clinician); Standard metrics/thresholds used (use Functional_assessment or Theme 4 triage codes)

##### Subtheme 2.2 – Outcome assessment

**Code:** Functional_assessment

**Description:** Assessment of physical function during follow-up.

**Include when:** Range of motion, walking ability, strength, or mobility is discussedFunctional thresholds are mentioned (e.g., 90° flexion).

**Exclude when:** Function is assessed digitally (Theme 3)

**Code:** Wound_assessment

**Description:** Evaluation of surgical wound healing.

**Include when:** Wound leakage, redness, infection are discussedStaple or suture removal is mentioned

**Exclude when:** Wound images are sent digitally (Theme 4)

**Code:** Pain_assessment

**Description:** Evaluation of pain.

**Include when:** pain control, levels or management are discussed; analgesia appliance is noted

**Exclude when:** pain levels are assessed digitally

**Code:** Patient_walking_observation

**Description:** Specific statements about observing the patient walking (often as they enter or in the waiting room) as a key informational cue.

**Include when:** “I see them walk in the waiting room” or equivalent is stated; Walking-in observation is framed as giving immediate impression.

**Exclude when:** General statements about mobility or function without this observational moment (use Mobility_observation)

**Code:** Mobility_observation

**Description:** Clinician references to observing mobility (general gait, transferring, using aids) as part of assessment.

**Include when:** Observation of walking ability, mobilizing with/without crutches, functional movement is described.

**Exclude when:** Specifically observing how patient walks into room/waiting area (use Patient_walking_observation); Formal ROM measures or tests are described without observational emphasis (use Functional_assessment)

##### Subtheme 2.3 – Reassurance and relational value

**Code:** Xray_as_reassurance

**Description:** Imaging is used communicatively/educationally during the visit.

**Include when:** Xray is used for reassurance understanding reasons.

**Code:** Visit_for_reassurance

**Description:** Outpatient contact is framed as preventing downstream care by providing reassurance/clarity.

**Include when:** Highlights reassurance/relationship as an important (non-medical) function of visits.

**Code:** Added_value_of_outpatient_visit

**Description:** Positions part of the outpatient encounter as informational/reassurance-oriented.

**Include when:** this is discussed beyond pure clinical metrics.

**Code:** Visit_as_tradition

**Description:** Statements that a visit (or imaging) is done mainly because it has historically been done, rather than because of clear clinical need.

**Include when:** “because we always did it”, “tradition”, “habit”; Visit framed as customary.

**Exclude when:** Justified by guidelines (use Guideline_influenced_practice); Justified by reassurance or expectations (use Visit_for_reassurance / Added_value_of_outpatient_visit)

**Code:** Patient_anxiety_or_uncertainty

**Description:** References to patient worry, uncertainty, reassurance-seeking, or needing confirmation that recovery is okay.

**Include when:** Patients feel safer with a visit; The follow-up visit functions to reduce anxiety.

**Exclude when:** General reassurance value of visits without explicit anxiety framing (use Visit_for_reassurance); Anxiety linked to digital literacy barriers (use Health_literacy_issues / Digital_exclusion)

#### Theme 3 – Digital follow-up conceptualized as triage mechanism

##### Subtheme 3.1 – Potential role and data sources

**Code:** Positive_attitude_digital_followup

**Description:** Explicit support for digital follow-up.

**Include when:** Benefits such as efficiency or access are highlighted

**Exclude when:** Support is conditional (Theme 4)

**Code:** Neutral_attitude_digital_followup

**Description:** Ambivalent or cautious openness toward digital follow-up.

**Include when:** “Could work, but…” statements appear

**Exclude when:** Clear skepticism is expressed

**Code:** Negative_attitude_digital_followup

**Description:** Negativeness toward digital follow-up.

**Include when:** negative statements appear

**Exclude when:** Negativeness is conditional (theme 4)

**Code:** Reduced_patient_burden

**Description:** Excplicit notings about reduced patient burden or pressure on capacity on healthcare providers.

**Include when:** Patient burden reduction is a key benefit of avoiding routine visits.

**Code:** Efficiency_gain_digital_followup

**Description:** Excplicit notings about efficiency gain in providing care to patients in general.

**Include when:** efficiency gains are expolicitly mentioned

**Code:** Better_resource_allocation

**Description:** Excplicit notings about better resource allocation.

**Include when:** interviewee mentiones about appropriate care(-provider) at the appropriate place

**Code:** Skepticism_toward_sensors

**Description:** Concerns or doubts about sensors, wearable tech, or passive measurement approaches for follow-up.

**Include when:** Interviewee doubts feasibility/acceptance/validity of sensors or insole-type tools.

**Exclude when:** Critique is specifically about step counts or clinical relevance (use Steps_not_clinically_relevant); General skepticism about digital tools not specifically sensors (use Skepticism_toward_digital_tools)

**Code:** Wearables_not_useful

**Description:** Explicit statements that wearables/sensors add little value or are not useful in practice.

**Include when:** “not useful”, “no added value”, “too complex”, “patients won’t use it”.

**Exclude when:** Skepticism is tentative or conditional (use Skepticism_toward_sensors); The issue is equity/digital ability (Theme 4: Digital_exclusion)

**Code:** Steps_not_clinically_relevant

**Description:** Statements that step counts/activity tracking are not clinically meaningful indicators of knee recovery or decision-making.

**Include when:** Interviewee explicitly questions relevance of steps as outcome/trigger; Step count is framed as misleading or insufficient.

**Exclude when:** Steps/activity is proposed as useful proxy by clinician (that would be coded under Data_driven_decision_making or Patient_selection_criteria depending on context)

**Code:** Workforce_shortage

**Description:** References to current or anticipated shortages of orthopaedic staff driving redesign of follow-up.

**Include when:** Explicit mention of shortage of orthopaedists or staff capacity constraints

**Exclude when:** General efficiency or workload pressure without staffing shortage claim (use Efficiency_pressure)

**Code:** Efficiency_pressure

**Description:** Statements about pressure to increase efficiency, reduce clinic load, shorten waiting lists, or manage time scarcity.

**Include when:** “time is scarce”, “clinic is full”, “reduce waiting list”, “efficiency step”.

**Exclude when:** Pressure described as sustainability of healthcare system long-term (use Sustainability_of_care); Organizational infrastructure issues (use Organisational_readiness)

**Code:** Sustainability_of_care

**Description:** Statements framing digital follow-up as necessary for long-term sustainability of healthcare due to rising demand/costs.

**Include when:** “pressure on healthcare”, “future demand”, “keep care affordable”, “system sustainability”.

**Exclude when:** Immediate clinic efficiency pressures without long-term framing (use Efficiency_pressure)

##### Subtheme 3.2 – Perceived concerns and risks

**Code:** Skepticism_toward_digital_tools

**Description:** Concerns or doubts about digital follow-up.

**Include when:** Risk of missing complications is mentioned; Data reliability is questioned.

**Exclude when:** Concerns relate to governance rather than tools (Theme 4)

**Code:** Loss_of_personal_contact

**Description:** Perceived loss of relationship or presence due to digital follow-up.

**Include when:** Doctor–patient relationship is emphasized; Emotional or moral concerns are raised

**Exclude when:** Contact loss is logistical only

**Code:** Risk_of_missing_complications

**Description:** Concerns about missing complications when patients are not receiving in-person contact on site.

**Include when:** participant notes an increased likelyhood of missing complications; Emotional or moral concerns are raised

**Exclude when:** Contact loss is logistical only

**Code:** Digital_followup_not_for_everyone

**Description:** Concerns about missing excluding patients based on their digital skills.

**Include when:** participant notes an increased likelyhood of exluding patients from care based on digital skills; Emotional or moral concerns are raised

**Exclude when:** Contact loss is logistical only

**Code:** Skepticism_toward_sensors

**Description:** Concerns or doubts about sensors, wearable tech, or passive measurement approaches for follow-up.

**Include when:** Interviewee doubts feasibility/acceptance/validity of sensors or insole-type tools.

**Exclude when:** Critique is specifically about step counts or clinical relevance (use Steps_not_clinically_relevant); General skepticism about digital tools not specifically sensors (use Skepticism_toward_digital_tools)

**Code:** Wearables_not_useful

**Description:** Explicit statements that wearables/sensors add little value or are not useful in practice.

**Include when:** “not useful”, “no added value”, “too complex”, “patients won’t use it”.

**Exclude when:** Skepticism is tentative or conditional (use Skepticism_toward_sensors); The issue is equity/digital ability (Theme 4: Digital_exclusion)

**Code:** Steps_not_clinically_relevant

**Description:** Statements that step counts/activity tracking are not clinically meaningful indicators of knee recovery or decision-making.

**Include when:** Interviewee explicitly questions relevance of steps as outcome/trigger; Step count is framed as misleading or insufficient.

**Exclude when:** Steps/activity is proposed as useful proxy by clinician (that would be coded under Data_driven_decision_making or Patient_selection_criteria depending on context)

**Code:** Health_literacy_issues

**Description:** Statements that health literacy affects the ability to follow instructions, self-report, or engage with follow-up pathways (digital or otherwise).

**Include when:** “health literacy”, understanding questions, ability to interpret symptoms, adherence issues; Often overlaps with selection criteria.

**Exclude when:** Specifically about digital skills/access (use Digital_exclusion); General patient selection without literacy dimension (Low_risk_patients)

**Code:** Digital_exclusion

**Description:** Statements about patients being unable or unlikely to use digital tools due to age, education, digital skills, access, or other barriers.

**Include when:** “not digitally skilled”, “older”, “education level”, “won’t use an app; ”Digital inequality is mentioned

**Exclude when:** Broad equity discussion without digital focus (use Equitable_access_to_care)General platform usability complaints (Theme 3: Skepticism_toward_digital_tools)

**Code:** Equitable_access_to_care

**Description:** Statements emphasizing fairness and ensuring access for all patients regardless of ability or resources.

**Include when:** Concern that digital pathways should not disadvantage vulnerable groups; Discussion about maintaining alternatives and open access.

**Exclude when:** Practical barrier of not using digital tools (use Digital_exclusion); Patient choice to come in (use Patient_preference_physical_visit or Clinical_override_protocol)

**Code:** Language_barriers

**Description:** Criteria used to determine who who has a language barrier.

**Include when:** language is mentioned

**Exclude when:** General population-level statements

#### Theme 4 – Acceptance of digital follow-up is conditional

##### Subtheme 4.1 – Patient selection

**Code:** Low_risk_patients

**Description:** Statements identifying a subset of patients considered suitable for reduced/digital follow-up due to low risk or straightforward recovery.

**Include when:** “simple cases”, “uncomplicated”, “low risk” as eligibility; Criteria may be implicit but patient group is clearly described.

**Exclude when:** Explicit thresholds (ROM/pain/wound) are detailed (use Patient_selection_criteria); Equity barriers are discussed (use Digital_exclusion / Health_literacy_issues)

**Code:** High_risk_patients

**Description:** Statements identifying higher-risk patients who require more intensive or in-person follow-up.

**Include when:** “high risk”, “comorbidities”, “complex”, “revision”, “complications” as group not suited for digital-only.

**Exclude when:** The point is “complex cases need physical visit” (use that code when explicit); The statement is about deviations emerging after surgery (use Deviating_recovery)

**Code:** Complex_cases_need_physical_visit

**Description:** Explicit statements that complex cases or certain clinical situations require in-person assessment and cannot be managed digitally.

**Include when:** “must be seen physically”, “can’t leave to computer”, “need hands-on exam”, “need imaging” framed as non-negotiable.

**Exclude when:** Preference rather than necessity is expressed (use Patient_preference_physical_visit or Loss_of_personal_contact); Complexity is described without linking to necessity of in-person visit (High_risk_patients)

**Code:** Patient_selection_criteria

**Description:** Criteria used to decide who is eligible for digital follow-up.

**Include when:** Functional thresholds, risk profiles, or complexity are mentioned

**Exclude when:** Criteria are implicit rather than explicit

##### Subtheme 4.2 – Clinical decision rules

**Code:** Data_driven_decision_making

**Description:** Statements that decisions about visits should be guided by structured data, thresholds, or decision rules (e.g., “green/yellow/red”, ROM thresholds, pain thresholds).

**Include when:** Explicit triage criteria or thresholds are defined; Mention of flags, rule-based scheduling/cancellation.

**Exclude when:** Decisions rely on clinician intuition (use Gut_feeling_clinician); Data collection is mentioned without decision linkage (Theme 3: Virtual_care_platform or PROMs_based_followup)

**Code:** Questionnaire_based_assessment

**Description:** Use of questionnaires in follow-up.

**Include when:** questionnaires and their answers are mentioned for deciding whether a digital follow-up is indicated; Benefits or limits are discussed.

**Exclude when:** validated PROMs are used only for research or benchmarking

**Code:** Photo_or_videographic_assessment

**Description:** Use of photo- and videographic data in follow-up.

**Include when:** photo- and videographic assessment is mentioned for wound healing or walking/mobility observation for deciding whether a digital follow-up is indicated; Benefits or limits are discussed.

**Exclude when:** wounds or walking/mobility is observed during a visit.

**Code:** Sensor_or_wearable_assessment

**Description:** Use of sensor or wearable data in follow-up.

**Include when:** sensor or wearable data is mentioned for deciding whether a digital follow-up is indicated; Benefits or limits are discussed.

**Exclude when:** sensor or wearable data is standard care or implemented for research purposes

**Code:** Alarm_symptoms

**Description:** Signs or symptoms that trigger concern or additional follow-up.

**Include when:** Pain increase, loss of function, infection signs mentioned

**Exclude when:** General recovery complaints without concern

##### Subtheme 4.3 – Patient and clinician autonomy

**Code:** Patient_preference_physical_visit

**Description:** Respect for patient desire to be seen in person.

**Include when:** Patient comfort or preference determines visit

**Exclude when:** Preference is overridden by protocol

**Code:** Trust_in_patient_self_management

**Description:** Reliance on patients to initiate contact when needed.

**Include when:** “Door is always open” logic is expressed

**Exclude when:** Trust is conditional on digital monitoring

**Code:** Patient_preference_digital_followup

**Description:** Statements that patients prefer digital/remote follow-up, or benefit from avoiding travel/visits.

**Include when:** Patient convenience, time-saving, preference for not coming in; Examples of patients questioning necessity of visits.

**Exclude when:** Patient preference for in-person follow-up (use Patient_preference_physical_visit); Patient anxiety requiring reassurance (use Patient_anxiety_or_uncertainty)

**Code:** Clinical_override_protocol

**Description:** Mechanisms allowing clinicians to overrule digital pathways.

**Include when:** Escalation, opt-out, or discretionary review is described.

**Exclude when:** Override is purely patient-driven

**Code:** Professional_autonomy

**Description:** Protection of clinician decision-making authority.
**Include when:** Control, responsibility, or ownership is emphasized

**Exclude when:** Autonomy refers to organizational policy only

**Code:** Importance_of_clinical_judgement

**Description:** Statements asserting that human clinical judgment is essential and cannot be fully replaced by algorithms, PROMs, or protocols.

**Include when:** “cannot leave to computer”, “doctor must judge”, “open questions need clinician”, “some things cannot be automated”.

**Exclude when:** Emphasis is on relational aspect rather than judgment (Theme 3: Loss_of_personal_contact); Discussion is about override mechanisms specifically (use Clinical_override_protocol)

**Code:** Organisational_readiness

**Description:** Statements about infrastructural, workflow, governance, staffing, or coordination requirements needed to implement digital follow-up.

**Include when:** Need for integration with EHR, project lead, coordination, workflow ownership“; infrastructure crucial”, “must be organized”, “someone must monitor”.

**Exclude when:** Evidence requirements (use Proof_before_implementation); Purely clinical decision rule discussion (use Data_driven_decision_making)

### 4. Cross-case saturation matrix

Below is a **condensed cross-case saturation matrix.** It demonstrates **analytic saturation** (presence, consistency, and redundancy), not mere frequency.

| **Interview ID** | **Theme 1 Fragmented Routine Follow-up regimens between care settings** | **Theme 2 Various values of follow-up visits** | **Theme 3 Digital follow-up conceptualized as triage mechanism** | **Theme 4 Acceptance of digital follow-up is conditional** |
| --- | --- | --- | --- | --- |
| 1 | ● | ● | ● | ● |
| 2 | ● | ● | ● | ● |
| 3 | ● | ● | ○ | ○ |
| 4 | ● | ● | ● | ● |
| 5 | ● | ● | ● | ● |
| 6 | ● | ● | ● | ● |
| 7 | ● | ● | ○ | ○ |
| 8 | ● | ● | ○ | ○ |
| 9 | ● | ● | ● | ● |
| 10 | ● | ● | ○ | ○ |
| 11 | ● | ● | ● | ● |
| 12 | ● | ● | ● | ● |
| 13 | ● | ● | ● | ● |
| 14 | ● | ● | ● | ● |
| 15 | ● | ● | ● | ● |
| 16 | ● | ● | ● | ● |
| 17 | ● | ● | ● | ● |
| 18 | ● | ● | ● | ● |
| 19 | ● | ● | ● | ● |
| 20 | ● | ● | ● | ● |
| 21 | ● | ● | ● | ● |

**● = strongly present** (multiple explicit statements); **○ = present** (clear but less elaborated)

### 5. Participant and interview characteristics

| **Interview ID** | **Sex** | **Type** | **Years of experience** | **Type of care-setting** | **Date of interview** | **Interview duration** |
| --- | --- | --- | --- | --- | --- | --- |
| 1 | M | Orthopedic surgeon | 2 | Non-academic teaching | June 19, 2025 | 41 min |
| 2 | M | Orthopedic surgeon | 5 | General | June 24, 2025 | 46 min |
| 3 | M | Orthopedic surgeon | 8 | Non-academic teaching | June 24, 2025 | 37 min |
| 4 | M | Orthopedic surgeon | 6 | General | June 25, 2025 | 28 min |
| 5 | M | Orthopedic surgeon | 14 | Non-academic teaching | June 30, 2025 | 48 min |
| 6 | M | Orthopedic surgeon | 4 | Private clinic | June 30, 2025 | 36 min |
| 7 | M | Orthopedic surgeon | 17 | Non-academic teaching | July 1, 2025 | 21 min |
| 8 | M | Orthopedic surgeon | 15 | Non-academic teaching | July 4, 2025 | 37 min |
| 9 | F | Orthopedic surgeon | 17 | General | July 14, 2025 | 38 min |
| 10 | M | Orthopedic surgeon | 20 | General | July 21, 2025 | 41 min |
| 11 | M | Orthopedic surgeon | 7 | Non-academic teaching | July 21, 2025 | 40 min |
| 12 | M | Orthopedic surgeon | 25 | Non-academic teaching | July 28, 2025 | 32 min |
| 13 | M | Orthopedic surgeon | 17 | Non-academic teaching | August 11, 2025 | 38 min |
| 14 | M | Orthopedic surgeon | 8 | Non-academic teaching | September 1, 2025 | 30 min |
| 15 | M | Orthopedic surgeon | 11 | Academic | September 2, 2025 | 32 min |
| 16 | M | Orthopedic surgeon | 5 | Private clinic | September 3, 2025 | 30 min |
| 17 | M | Orthopedic surgeon | 9 | General | September 11, 2025 | 42 min |
| 18 | M | Orthopedic surgeon | 8 | Non-academic teaching | September 16, 2025 | 39 min |
| 19 | M | Orthopedic surgeon | 19 | Non-academic teaching | October 21, 2025 | 37 min |
| 20 | M | Orthopedic surgeon | 8 | Academic | October 28, 2025 | 28 min |
| 21 | M | Orthopedic surgeon | 13 | Private clinic | November 18, 2025 | 35 min |
